# GFLNet: A lightweight real-time detector with knowledge-guided compression in remote sensing images

**DOI:** 10.1371/journal.pone.0361806

**Published:** 2026-10-30

**Authors:** Jing Zhang, Mas Rina Mustaffa, Fatimah Khalid, Zainal Abdul Kahar, Tao Zhang

**Affiliations:** 1 Faculty of Computer Science and Information Technology, Universiti Putra Malaysia, Serdang, Malaysia; 2 Department of General Education, Anhui Vocational College of City Management, Hefei, China; 3 Whole vehicle development-Material technology, Volkswagen China Technology Company, Hefei, Anhui, China; Northeastern University, CHINA

## Abstract

Remote sensing images usually exhibits densely distributed targets and scale disparities. In such scenarios, it is still challenging to preserve high detection precision while controlling costs and inference latency. To address this issue, we propose GFLNet, a lightweight single-stage detector, which is based on collaborative feature modeling and knowledge-guided compression. Specifically, Ghost-Shuffle dual fusion convolution (GSD-Fuse) is designed to achieve efficient spatial-channel interaction while reducing computational cost; an adaptive receptive-field selection module inspired by LSKNet is introduced into the deeper backbone and neck stages to adaptively adjust contextual responses for small-object and multi-scale detection; finally, LB-Compress combines layer-adaptive magnitude pruning with Bidirectional Cross-task Knowledge Distillation (BCKD) to form a compact network suitable for practical deployment. Evaluated on the DIOR and UCAS-AOD datasets, GFLNet improves mAP@0.5 over the baseline by 3.9% and 3.4%, respectively. Moreover, the model contains only 2.35M parameters, with a calculation of 5.6 GFLOPs, and an inference speed of 261 FPS on the GPU, thereby delivering a favorable accuracy-efficiency synergy.

## 1 Introduction

Remote sensing object detection (RSOD) is vital in agriculture, traffic management, and emergency rescue [1]. Recently, CNN-based deep learning technology has been widely used to improve detection performance. Existing detectors can be divided into two-stage detectors and single-stage detectors. Two-stage detectors, including Fast R-CNN [2], Faster R-CNN [3], and Mask R-CNN [4], generate candidate areas, and then classify and border box regression. These methods usually have high accuracy, but the candidate area generation stage increases the calculation overhead and inference delay. One-stage detectors, including the YOLO series [5–7], SSD [8], and RetinaNet [9], perform classification and localization in a single forward pass. They are therefore well suited to real-time applications.

However, many recent studies have focused mainly on improving detection accuracy. This often increases model complexity and computational cost. Consequently, advanced detectors can be difficult to deploy on airborne platforms, environmental monitoring systems, and edge devices with limited resources. Lightweight networks such as MobileNet and ShuffleNet reduce computation through efficient operations [10,11]. Pruning, quantization, and knowledge distillation can further compress detection models [12–14]. However, remote sensing images (RSIs) usually contain densely distributed small objects, significant scale changes, arbitrary directions and complex backgrounds [15]. These features require the detector to capture enough contextual information while retaining spatial details. Increasing network depth, feature width, attention complexity or feature-aggregation stages usually increases computational cost, memory consumption and inference latency. Conversely, excessive architectural simplification or compression may weaken channel interaction and remove features critical to small-object detection.

The existing lightweight RSOD methods address these challenges through a compact backbone, multi-scale feature enhancement, attention mechanisms, and optimization strategies. YOLO-TLA and LMSD-YOLO enhance the multi-scale representation of small targets or SAR ship detection [16,17]. GCL-YOLO, CSPPartial-YOLO and YOLO-Lite reduce computational complexity through lightweight structural design [18–20]. Other methods improve aerial detection by refining YOLOv8, introducing GSConv, combining GhostBlockV2 with WiseIoU, or preserving spatial details through SPD-Conv and attention mechanisms [21–24]. However, these techniques are usually developed as independent optimization strategies. Simplified operators may limit spatial-channel interaction, fixed receptive fields may not be able to adapt to substantial object-scale variation, and the compression methods applied alone may lead to the loss of semantic and localization information. Therefore, accuracy recovery through feature interaction, adaptive context selection and compression should be considered in a unified detection framework.

Based on this analysis, lightweight RSOD still presents three challenges. First, simplified operators can weaken the cross-channel interaction and small-object representation. Second, the fixed receptive field and the limited multi-scale interaction may be insufficient for large-scale variation and dense backgrounds. Third, pruning and knowledge distillation are often implemented as separate post-processing procedures, which may result in information loss and accuracy degradation at high compression ratios. To solve these problems, GFLNet combines lightweight feature enhancement with model compression. GSD-Fuse strengthens spatial–channel interaction, and the LSKNet-inspired receptive field selection technique improves the scale–adaptive context modeling. The main contributions are listed below:

(1) GSD-Fuse for efficient feature fusion. We propose GSD-Fuse, a lightweight convolutional fusion module that strengthens spatial-channel interaction with reduced computation, improving feature representation for small objects in RSIs while avoiding repeated use of full standard convolutions.
(2) LSKNet-inspired adaptive receptive-field integration. We integrate an LSK mechanism, which adaptively adjusts receptive fields according to local content to enhance discrimination under complex backgrounds and multi-scale variations, into the deep backbone and neck to improve multi-scale semantic representation with limited overhead. We present ARSK,
(3) LB-Compress and GFLNet. We introduce LB-Compress, which combines layer-adaptive pruning with cross-task knowledge distillation. on DIOR and UCAS-AOD, our method attains a 3.9% and 3.4% improvement in mAP@0.5, with 2.35M parameters, 5.6 GFLOPs, a 5.2 MB model size, and 261 FPS on GPU.

The remainder of this article is organized as follows. The Related Work section reviews lightweight object detection and model compression, lightweight remote sensing detection, and recent studies on collaborative feature interaction and context modeling. The methodology section elaborates the GFLNet framework in detail, covering the overall network structure, the LSK module, the GSD-fusion module, and the LB-Compress strategy combining layer adaptive pruning and knowledge distillation. The experimental part introduces the datasets, implementation details, evaluation indicators, comparison experiments, ablation experiments and visualization results. the final section concludes major findings, followed by limitations and future exploration.

## 2 Related work

### 2.1 Lightweight object detection and model compression

The compact object detector is mainly realized through efficient architecture design and model compression. The architectural design route adopts a more economical alternative to replace expensive intensive computing. Howard et al. [10] proposed MobileNet, which uses deep separable convolution to decouple spatial filtering with channel mixing. Zhang et al. [11] introduced ShuffleNet to facilitate the exchange of information between group convolutions through channel rearrangement. Although both designs greatly reduce the amount of calculation, aggressive separation or grouping operations may weaken the communication between features and make weak visual evidence more difficult to characterize effectively.

Another line of research compresses an already trained network. Han et al. [12] reduced redundant connections through pruning, Jacob et al. [13] studied low-precision quantization for efficient integer-arithmetic inference, and Hinton et al. [14] introduced knowledge distillation, in which a compact student network learns from a larger teacher network. These methods can reduce model size and inference costs. However, applying compression independently may weaken features which preserve fine spatial detail. This is especially difficult for small objects, which have a small number of highly informative features.

### 2.2 Lightweight remote sensing detection

RSIs pose distinct challenges because of the small, densely packed, arbitrarily oriented objects, and surrounding background. In order to deal with these issues, several methods have been proposed. Wang et al. [15] proposed DSA-Det to handle extreme scale variation and small objects in optical remote sensing imagery. Li et al. [16] designed YOLO-TLA by combining multiscale feature enhancement with knowledge distillation. Guo et al. [17] proposed LMSD-YOLO for multi-scale SAR ship detection. Cao et al. [18] proposed GCL-YOLO by introducing GhostConv to reduce computational cost on UAVs. Xie et al. [19] simplified the CSP structure in CSPPartial-YOLO to obtain a lightweight detector for typical objects in RSIs. Ren et al. [20] developed YOLO-Lite for efficient SAR ship detection. These methods verify the effectiveness of compact structures, but when feature extraction is oversimplified, this simplification may weaken cross-channel information exchange.

Recent research has further improved the performance of lightweight aerial detection through architectural optimization strategies. Wang et al. [21] proposed YOLOv8 to improve small-object detection in low-altitude aerial imagery. Li et al. [22] designed GSConv, which combines three convolution types along with channel shuffle to strengthen feature interaction. Wang et al. [23] developed a small-object detector for RSIs based on CPDD-YOLOv8. Zhai et al. [24] further incorporated SPD-Conv and GAM attention into SPD-YOLOv8 to retain fine-grained spatial information in complex UAV scenes. Despite this progress, most of the existing methods optimize a single component, and do not explicitly coordinate feature interaction, adaptive context modeling, and the recovery of information lost during compression.

### 2.3 Lightweight remote sensing detection

Related remote sensing research indicates that effective representation learning depends not only on lightweight computation, but also on the joint modeling of spatial, scale, contextual, and channel information. Zhang et al. [25] proposed SGDFuse to preserve structural and semantic information in infrared and visible images. Xu et al. [26] combines multi-scale attention with adaptive guidance to improve change characterization. Ren et al. [27] designed a dual-attention-guided multiscale feature aggregation network to strengthen feature fusion for RSOD. Similar phenomena are also seen in semantic segmentation studies. Zhao et al. [28] proposed RGD-UNet with an efficient attention design to improve feature representation while maintaining relatively low computational cost. Fu et al. [29] introduced HyperR3SNet, which combines hyperbolic representations with vision models for semantic segmentation. Although these studies are aimed at different remote sensing tasks, these works collectively support joint modeling of multi-dimensional cues. Inspired by this observation, GFLNet coordinates space-channel interaction, adaptive field selection and compression strategy within a unified lightweight detection framework.

Although the above studies are aimed at different remote sensing tasks, they together show that spatial information, scale information, context information and channel information should be modeled collaboratively. Inspired by this, GFLNet coordinates efficient space-time channel interaction, adaptive sensory field selection and compressed perceptual knowledge recovery within a unified lightweight detection framework.

## 3 Methodology

### 3.1 Overview of the GFLNet

While current lightweight detectors have developed rapidly, they still perform poorly in RSIs. Key issues include insufficient feature representation, restricted multi-scale adaptability, and independent compression strategies. Although simplified operators reduce computation, they may sacrifice spatial details. The rigid sensory field cannot cover the large-scale change range of RSIs. To address these limitations, we propose GFLNet, a lightweight framework for RSOD. As shown in Fig 1, GFLNet uses a compct single-stage detector and integrates GSD-Fuse, LSK, and LB-Compress. GSD-Fuse improves feature interaction, LSK adapts contextual information to object scale, and LB-Compress reduces model size. Together, these modules achieve cost-effective detection without sacrificing accuracy.

**Fig 1:**
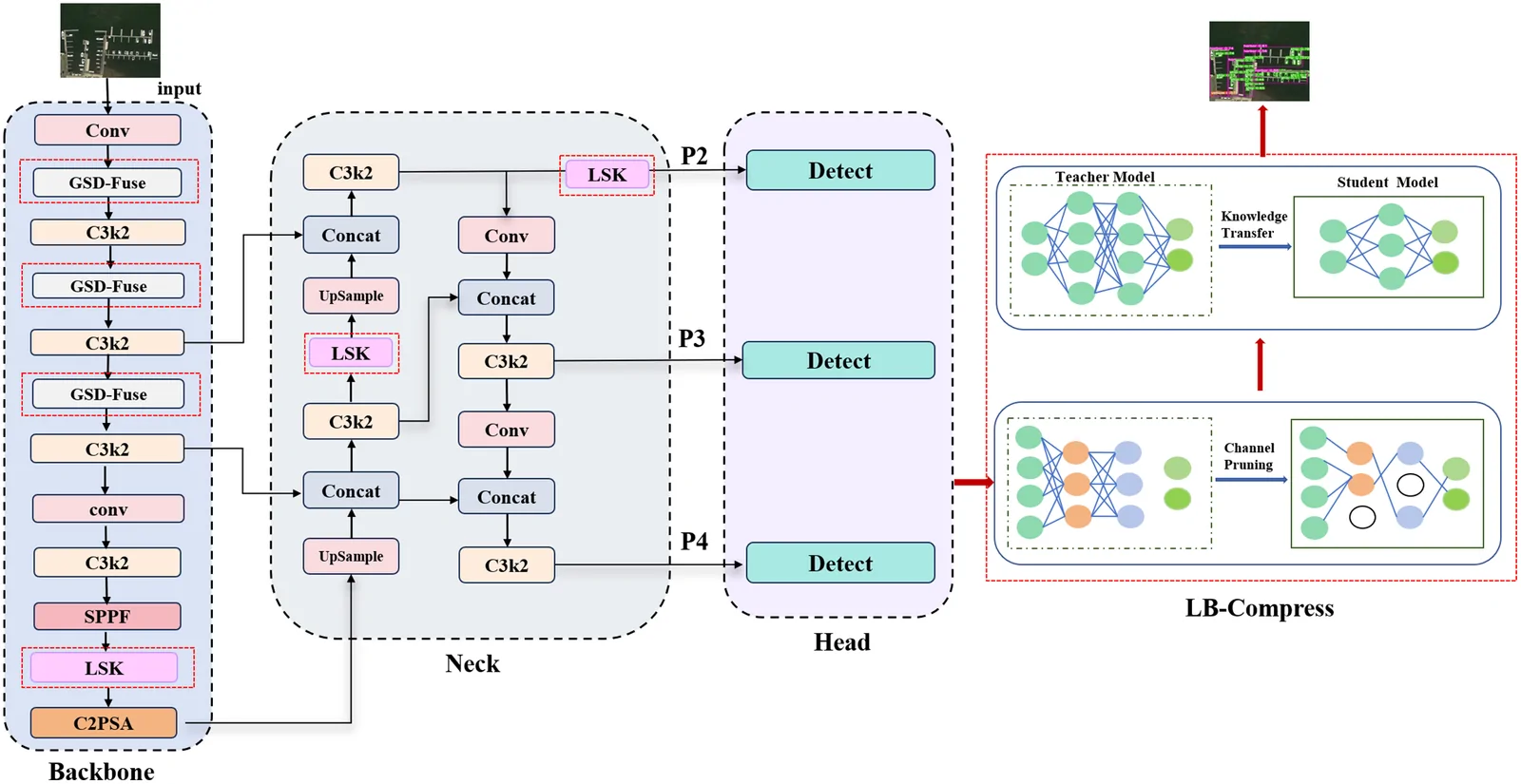
The global overview of the GFLNet.

To enhance the limited feature representation capacity caused by simplified operators, conventional convolutions in the shallow backbone are replaced with GSD-Fuse. It improves the ability of spatial interaction and channel interaction with only a small computational cost. In the deeper backbone network stage, LSK is introduced and further connected to the feature aggregation process in the neck network. Its receptive field mechanism enables the network to capture wider contextual information of large targets while retaining local details of small objects. Finally, LB-Compress combines layer adaptive pruning and knowledge distillation to reduce the size of the model and alleviate the decrease in accuracy. By embedding the compression process into the network optimization, GFLNet can achieve reliable multi-scale target detection under a limited computing budget.

### 3.2 LSK-inspired adaptive receptive-field module

The design motivation of the framework is to capture fine spatial details and rich contextual information to improve detection performance. Targets in RSIs usually have the characteristics of small size and complex background. When the long-distance context information is insufficient, the model is prone to omission or misclassification. In order to solve this problem, the Large Selective Kernel (LSK) module [30] introduces the dynamic receptive field mechanism. This module uses decomposed large-kernel convolution to extract multi-scale features. Meanwhile, the spatial selection method is proposed to automatically select the most useful contextual regions. The LSK module is able to extend the receptive field and allows content-aware adaptation, which greatly improves the robustness of the model to background interference and scale changes.

As illustrated in Fig 2, the LSK module constructs multi-receptive-field representations through a sequence of depthwise convolution operations. Different convolution kernels capture local details and broader contextual cues, while a spatial selection mechanism assigns higher weights to informative regions. For dense small objects, this adaptive selection preserves discriminative local details and suppresses background clutter. For large-scale variations, it allows the detector to adjust contextual ranges without increasing the computational overhead to every layer. The module first applies 5 × 5 and 7 × 7 depthwise convolutions to extract multi-scale contextual features. It then uses 1 × 1 convolutions $F_{i}^{1\times 1}$, and concatenates the resulting features $\tilde{U}=[\tilde{U_{\text{1}}};\tilde{U_{\text{2}}}]$ along the channel dimension. For the i-th convolution, the receptive field can be expressed as

**Fig 2:**
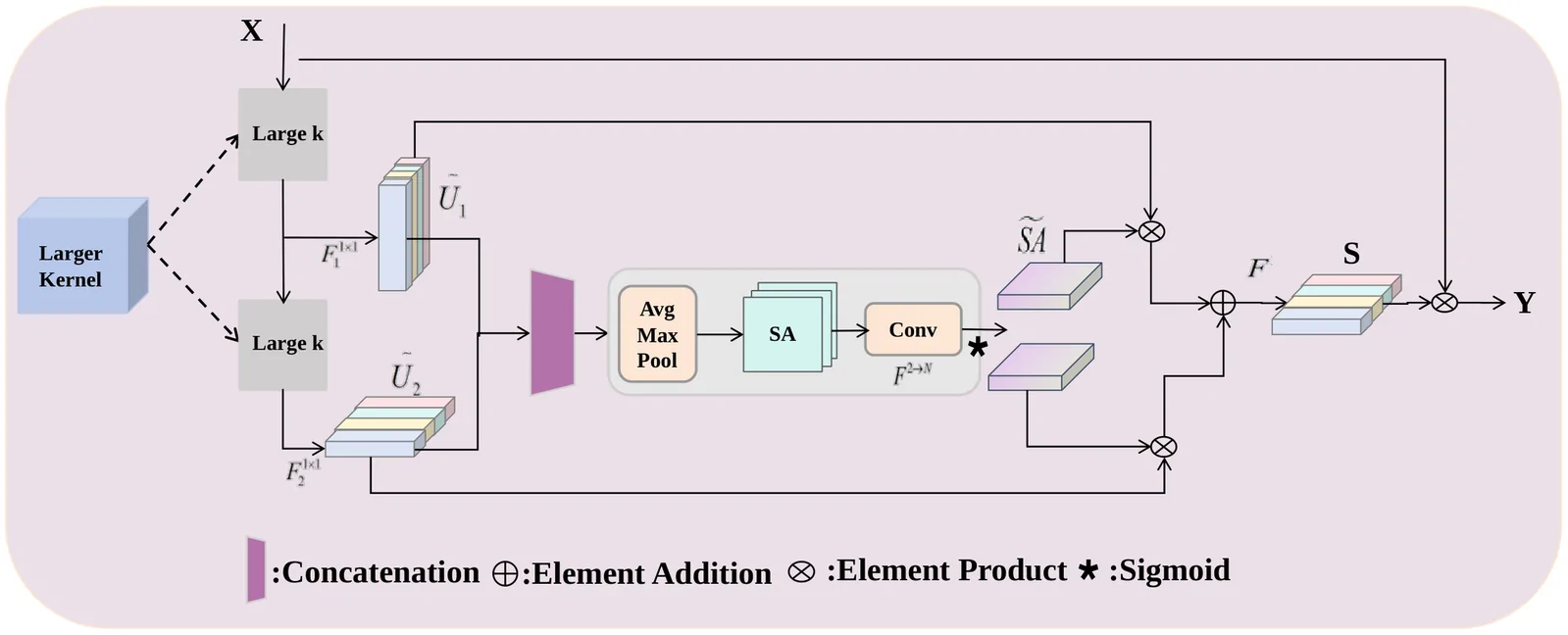
The LSK module.


$$\begin{array}{c} RF_{i}=RF_{i-1}+d_{i}(k_{i}-1) \end{array}$$ (1)

Where $k_{i}$ and $d_{i}$ denote the kernel size and dilation rate, respectively. The 5 × 5 kernel is used to retain local spatial details, while the 7 × 7 kernel provides a wider contextual range.

Spatial autocorrelation descriptors, denoted as ***SA***, are then generated through parallel average pooling and max pooling operations.


$$\begin{array}{c} SA_{\text{a}vg}=P_{avg}\left(\tilde{U}\right) \end{array}$$ (2)

$$\begin{array}{c} SAmax=P_{max}\left(\tilde{U}\right) \end{array}$$ (3)

$$\begin{array}{c} SA=[SA_{\text{a}vg},SAmax] \end{array}$$ (4)

The aggregated pooling features are further transformed by sigmoid function σ(·) and convolutional transformation $F^{2\to N}$ to obtain attention weights ***N***.


$$\begin{array}{c} N=\sigma \left(F^{2\to N}\left(SA\right)\right) \end{array}$$ (5)

The contextually enhanced representation is obtained through a weighted fusion of multi-scale features with their corresponding spatial attention weights, followed by a convolutional operation ***F*** that performs channel integration and dimensionality trade-off, outputting the refined contextual representation ***S***.


$$\begin{array}{c} S=F\left(\sum_{i=1}^{N}N_{i}\cdot \tilde{U_{i}}\right) \end{array}$$ (6)

The final output is obtained through element-wise modulation of the input features:


$$\begin{array}{c} Y=X⊙S \end{array}$$ (7)

In this way, the LSK module achieves efficient multi-scale context modeling and spatial adaptation, enhancing RSOD performance through emphasized feature selection and noise suppression.

### 3.3 Ghost-shuffle dual-fusion convolution

Shuffle convolution (SC) is the fundamental feature extraction operation in convolutional neural networks. This connected approach is able to capture the spatial information and relationship between channels, but the cost is more. To reduce this cost, depthwise separable convolution (DSC) is divided into channel-wise spatial filtering and pointwise convolution. Despite its efficiency, the early separation of channel information in DSC may weaken the interaction and limit its representation capacity. The structure of SC and DSC is compared in Fig 3.

**Fig 3:**
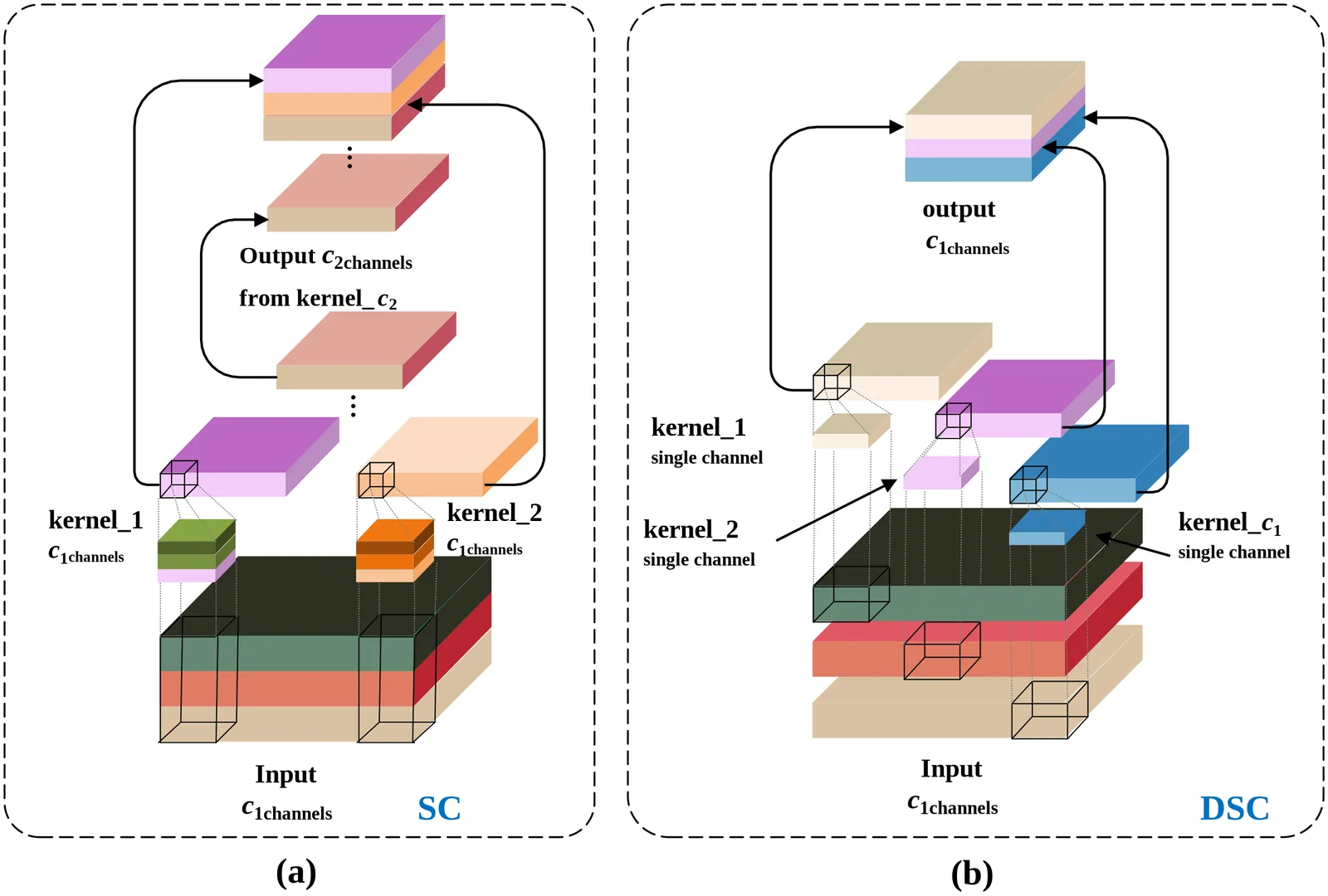
The module of (a) SC and (b) DSC.

To reduce the computational burden while preserving feature representation, the GSConv [22] adopts a dual-branch architecture that combines SC and DSC. As shown in Fig 4, the SC branch is used to retain stronger channel-wise representation, whereas the DSC branch provides a lightweight way to extract spatial information. In this design, each branch produces half of the output channels. The two outputs are then concatenated to recover the complete channel dimension. Channel shuffling is applied to boost cross-channel interactions. By combining the advantages of SC and DSC, GSConv improves feature representation with only a limited increase in computational cost.

**Fig 4:**
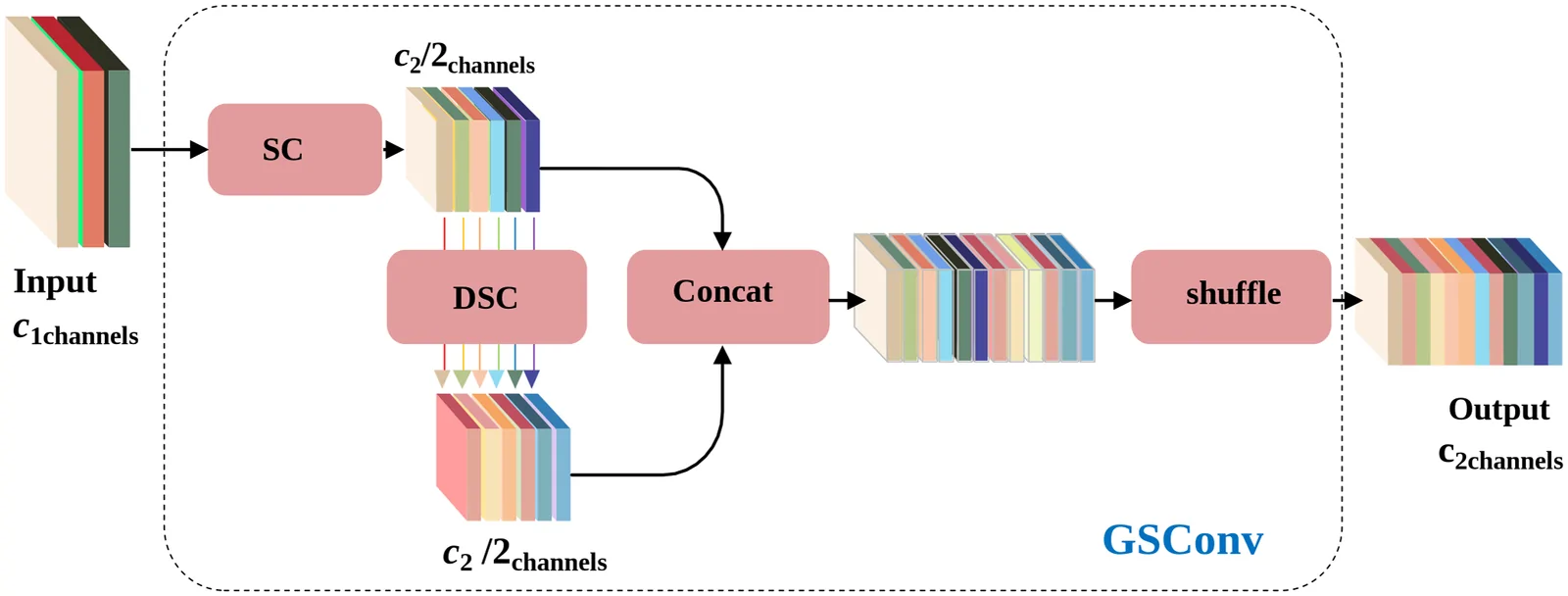
The structure of GSConv.

The Ghost–Shuffle Dual-Fusion Convolution (GSD-Fuse) is developed from GSConv to improve feature fusion efficiency. Unlike simply stacking GSConv layers, GSD-Fuse decouples channel representation and spatial feature enhancement into two cooperative branches. This structure preserves spatial sensitivity while maintaining the computational efficiency of GSConv. It also enables complementary feature modeling. The flow of GSD-Fuse is exhibited in Fig 5. The main branch uses two consecutive GSConv layers to extract channel and spatial information. Meanwhile, the residual branch retains part of the input features to limit information loss. The outputs of the two branches are concatenated and fused. Then, the outputs from both paths are merged and fused, and a 1 × 1 convolution reduces the channel dimension to produce the refined output features.

**Fig 5:**
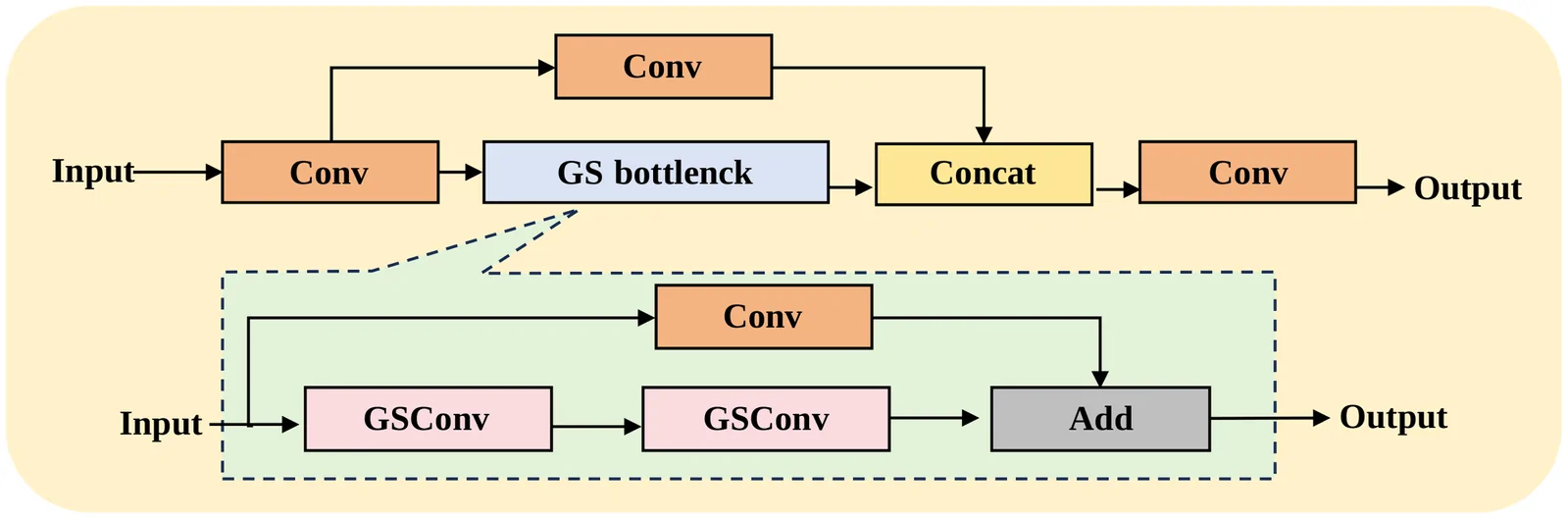
The components of GSD-Fuse.

To further compare the computational cost, let $C_{in}$ and $C_{out}$ denote the input and output channels, k denote the kernel size, and H×W denote the feature-map size. The parameter numbers of SC and DSC are


$$\begin{array}{c} P_{SC}=k^{\text{2}}C_{in}C_{out} \end{array}$$ (8)

$$\begin{array}{c} P_{DSC}=k^{\text{2}}C_{in}+C_{in}C_{out} \end{array}$$ (9)

GSConv combines standard and lightweight convolution branches, while GSD-Fuse further uses GSConv-based feature extraction and lightweight fusion. For a feature map of H×W, the FLOPs can be approximately calculated as


$$\begin{array}{c} F=2HWP \end{array}$$ (10)

This design reduces the repeated use of full standard convolutions and keeps the additional computational cost relatively low. Therefore, GSD-Fuse provides efficient feature fusion while keeping the additional computational cost relatively low.

### 3.4 LB-Compress

Although the above components improve the detection accuracy, the model still requires further compression for real-time deployment. LB-Compress combines layer-adaptive pruning and knowledge distillation. The two techniques work together: pruning removes redundant parameters, while distillation helps the compressed model recover information lost during pruning.

#### 3.4.1 Layer-adaptive magnitude-based pruning with learnable threshold.

Layer-Adaptive Magnitude-based Pruning (LAMP) [31] removes redundant parameters while maintaining a balanced distribution of sparsity across layers. Unlike conventional layer-wise pruning, LAMP evaluates all weights using a global importance score. This score enables direct comparison of weights across layers with different sensitivities. The pruning fraction is defined as follows:


$$\begin{array}{c} s_{i}=\frac{{w_{i}}^{\text{2}}}{\sum_{j\geq i}w_{j}^{\text{2}}} \end{array}$$ (11)

where $w_{i}$ denotes the i-th weight after global sorting in ascending order.

A learnable compression threshold τ is introduced to adapt the pruning process during training. It determines whether each weight is retained or removed:


$$\begin{array}{c} {\hat{w}}_{i}=\left\{ \begin{array}{c} @c@0,\\ w_{i} \end{array}\right.\begin{array}{cc} & \begin{array}{c} s_{i}<\tau \\ s_{i}\geq \tau \end{array} \end{array} \end{array}$$ (12)

Here, the weights $s_{i}$ below the threshold τ are set to zero, which creates explicit sparsity. Unlike the fixed pruning ratio, this method updates τ dynamically during training.


$$\begin{array}{c} \tau^{t+1}=\tau^{t}-\eta_{\tau }\frac{\partial L}{\partial \tau } \end{array}$$ (13)

Where $\eta_{\tau }$ denotes the learning rate of the threshold. This strategy distributes sparsity adaptively across layers and preserves those that are more sensitive to compression. Fig 6 compares the channel distribution before and after adaptive pruning. The results show that redundant channels are removed while essential network structures remain intact. This prevents excessive pruning during sensitive feature extraction stages.

**Fig 6:**
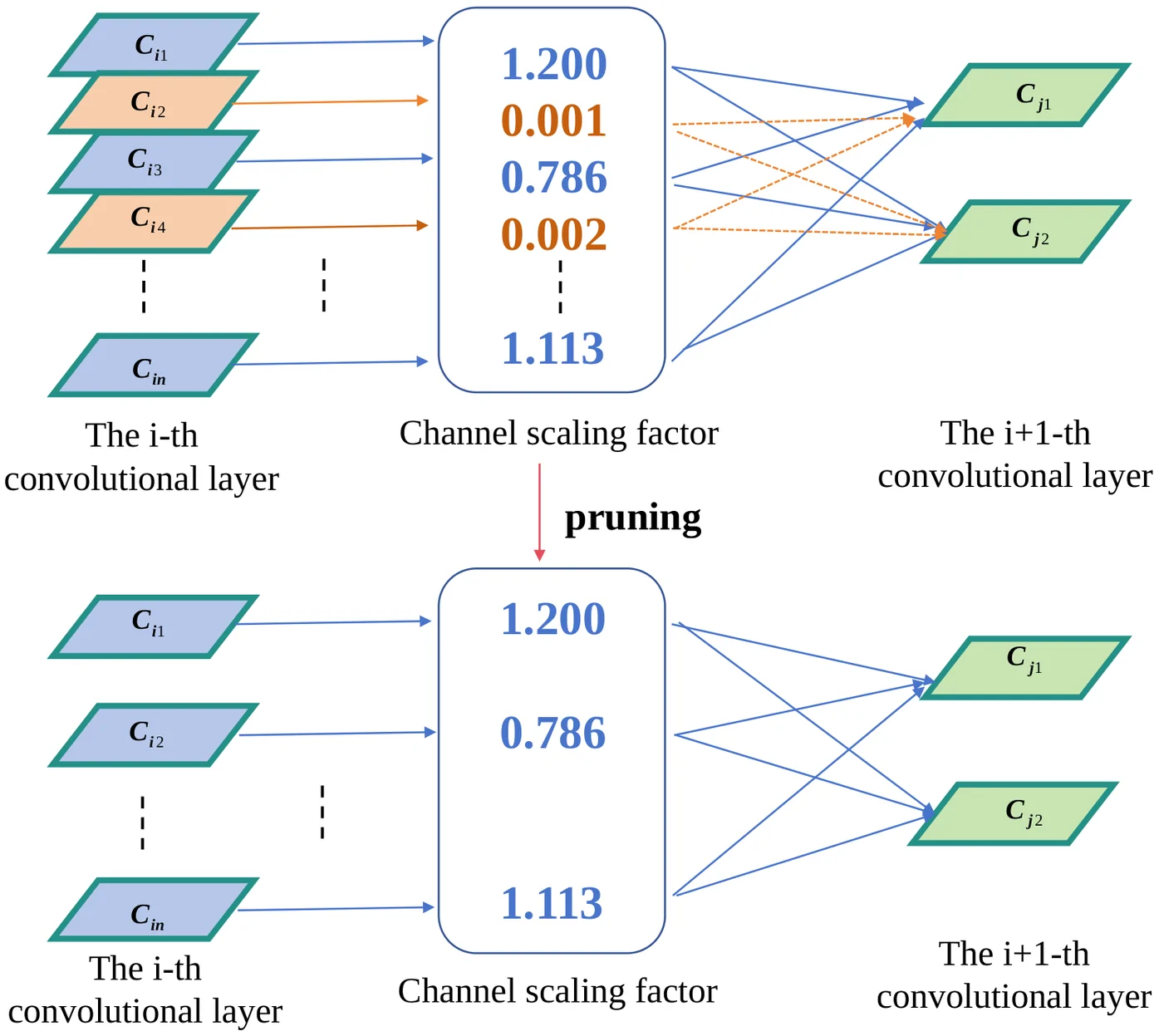
Schematic diagram of channel pruning process.

#### 3.4.2 Knowledge distillation as pruning compensation.

Pruning removes redundant weights but may also suppress useful feature responses, especially for small and densely distributed objects. To reduce this loss, we apply bidirectional cross-task knowledge distillation (BCKD) [32]. After pruning, the lightweight student detector learns classification and localization cues from a stronger teacher model. This supervision helps recover semantic discrimination and localization accuracy. BCKD is a logit-based method developed for dense target detection. It separates classification supervision from location supervision to solve the inconsistency between the two tasks.

The overall distillation objective comprises classification and localization guidance, as illustrated in Fig 7, jointly transferring category confidence and bounding-box alignment knowledge from the teacher to the pruned student model.

**Fig 7:**
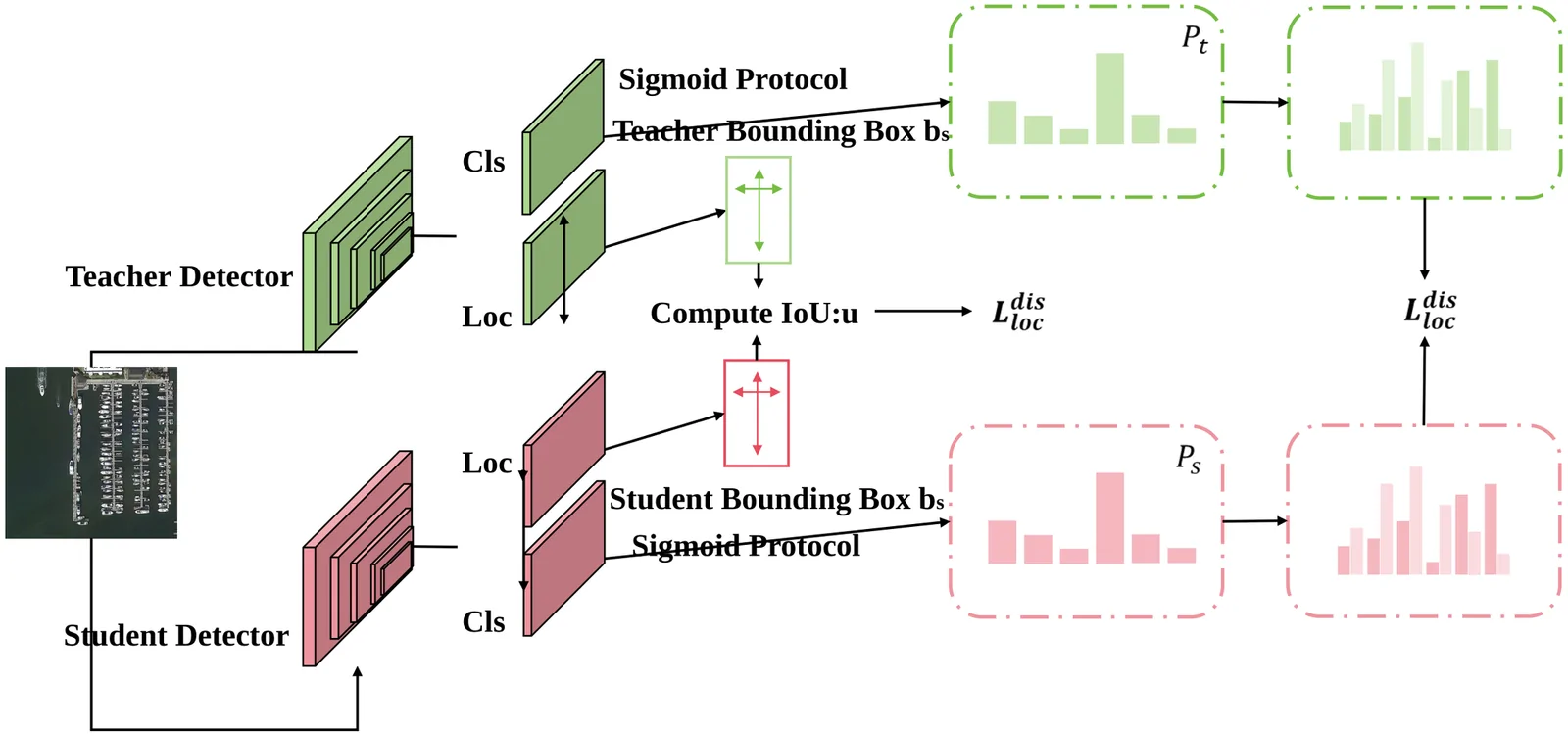
Overall architecture of BCKD knowledge distillation.

To handle class imbalance in dense detection, BCKD replaces Softmax-based multi-class distillation with Sigmoid-based binary supervision for each class independently:


$$\begin{array}{c} ProtSig=\frac{\text{1}}{1+e^{-l}} \end{array}$$ (14)

where l denotes the classification logit. The logit is converted into classification scores via the Sigmoid function, which effectively addresses the constraints of the Softmax protocol in dense detection.

The binary cross-entropy loss is then applied to align the student and teacher predictions:


$$\begin{array}{c} L_{BCE}\left(p_{i},y_{i}\right)=\left\{ \begin{array}{c} @l@-ln\left(p_{i}\right)\\ -ln(1-p_{i}) \end{array}\right.\begin{array}{cc} & \begin{array}{c} y_{i}=1\\ y_{i}=0 \end{array} \end{array} \end{array}$$ (15)

where $p_{i}$ denotes the classification score of the student model, and $y_{i}$ denotes that of the teacher model.

A discrepancy-dependent weight further adjusts the contribution of each teacher–student pair:


$$\begin{array}{c} w_{i,j}=\|{p^{\prime }}_{t,i,j}-{p^{\prime }}_{s,i,j}\| \end{array}$$ (16)

where the superscripts t and s refer to the teacher and student models, respectively. The quantities ${p^{\prime }}_{t,i,j}$ and ${p^{\prime }}_{s,i,j}$ are their predicted scores for class j at spatial location i. The weighted classification distillation loss is:


$$\begin{array}{c} L_{clc}^{dis}=\sum_{i=1}^{n}\sum_{j=1}^{k}w_{i,j}\cdot L_{BCE}\left({p^{\prime }}_{t,i,j},{p^{\prime }}_{s,i,j}\right) \end{array}$$ (17)

BCKD further introduces an IoU-based localization distillation loss $L_{loc}^{dis}$. It directly computes the IoU between the bounding boxes predicted by the teacher and student models. The IoU loss then minimizes the difference between the calculated IoU and the target value of 1. Since this loss is independent of a specific network architecture, it can be applied to a wide range of detection models. The corresponding formula is presented as follows:


$$\begin{array}{c} {u^{\prime }}_{i}=IoU\left(b_{s}^{i},b_{t}^{i}\right) \end{array}$$ (18)

where $b_{s}^{i}$ and $b_{t}^{i}$ denote the i-th bounding boxes predicted by the student and teacher models, respectively. The formula for the localization distillation loss is:


$$\begin{array}{c} L_{loc}^{dis}=\sum_{i=1}^{n}maxw_{i,j}\cdot (1-{u^{\prime }}_{i}) \end{array}$$ (19)

The final overall distillation loss formula is presented as follows:


$$\begin{array}{c} L_{total}^{dis}=\alpha_{\text{1}}\cdot L_{cls}^{dis}+\alpha_{\text{2}}\cdot L_{loc}^{dis} \end{array}$$ (20)

where $\alpha_{\text{1}}$ and $\alpha_{\text{2}}$ are hyperparameters that balance the classification and localization distillation losses.

#### 3.4.3 Collaborative effect of LB-compress.

Sparse models after pruning tend to suffer from overfitting, while knowledge distillation provides the student model with more generalized information via the soft labels of the teacher model. This alleviates the overfitting of sparse model and enhances robustness of small object detection in complex scenarios. LB-Compress is able to obtain a compression-aware optimization instead of shrinking the model after training by combining adaptive pruning and knowledge distillation. Pruning reduces the redundancy of the structure, and distillation compensates the information loss. With the cooperative design, GFLNet has a strong detection performance at high compression ratio.

## 4 Experiments

### 4.1 Datasets

DIOR is a comprehensive RSOD benchmark, containing 23,463 images, and contains 23,463 images with 20 categories including vehicles, chimney, stadium and buildings. The dataset covers various geographical locations, imaging scenarios and sensor types, and therefore contains notable variance in target size, angle and scene complexity.

UCAS-AOD is a remote sensing benchmark for aerial-object and vehicle detection in aerial images, published by the Chinese Academy of Sciences. It includes 1,510 high resolution images and annotations for two categories of objects. The background covers various scenes including urban roads, airports and mountainous areas. The dataset provides both horizontal and oriented bounding-box annotations, which supports the evaluation of performance on arbitrarily oriented small objects in cluttered scenes.

RSOD remains challenging due to large scale variations, complex backgrounds, and changes in object orientation [33,34]. To reveal the common challenges in RSOD, Fig 8 presents the scale and position distribution of the two datasets. The overall scale distribution shows that small objects make up a large proportion of instances in both datasets. Since these objects occupy only a few pixels and often appear in dense clusters, they are easily confused with surrounding background regions. Moreover, small targets also appear together with much larger objects, which further increases the scale variation. These characteristics make small-object detection a major difficulty in RSOD and motivate GFLNet to strengthen fine-grained feature representation under a lightweight design.

**Fig 8:**
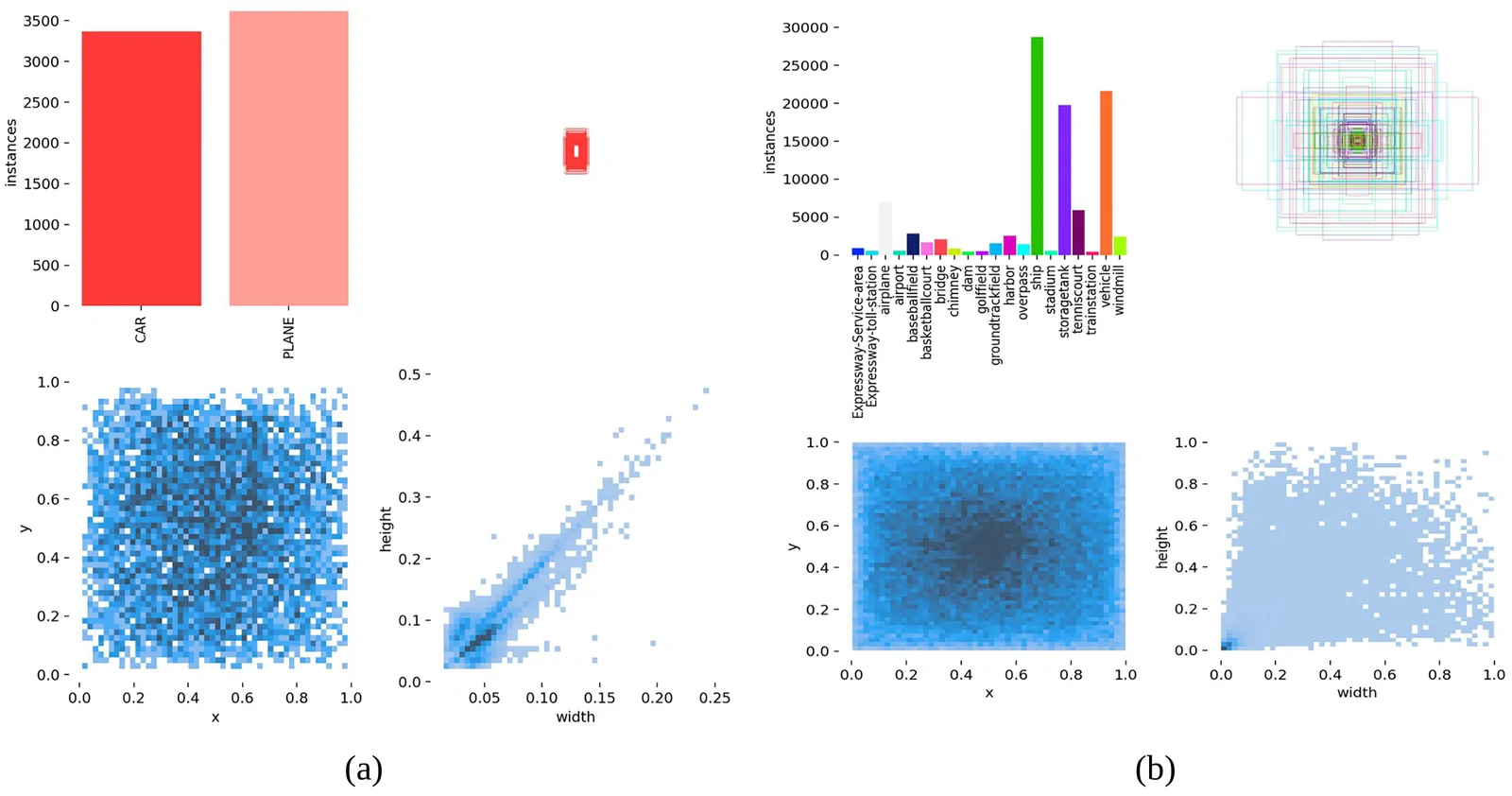
Distribution of target center locations and scales on UCAS-AOD (left) and DIOR (right).

### 4.2 Experimental environment

All experiments were done on Windows 10 laptop with NVIDIA RTX 4090 GPU, Python 3.8, PyTorch 1.10.0 and CUDA 11.3. The batch size of the training is 16 and the initial learning rate is 0.001. This is an order of magnitude that has been proposed to balance the training stability, convergence efficiency, and model accuracy. In order to be fair, all the reproduced models were trained in the same conditions. The result of some detectors is obtained from the original literature. In addition, edge-device inference was evaluated on an NVIDIA Jetson AGX Xavier. The input resolution was fixed at 800 × 800 with a batch size of 1, and the average latency was measured after warm-up. The training process of GFLNet on the UCAS-AOD dataset is shown in Fig 9.

**Fig 9:**
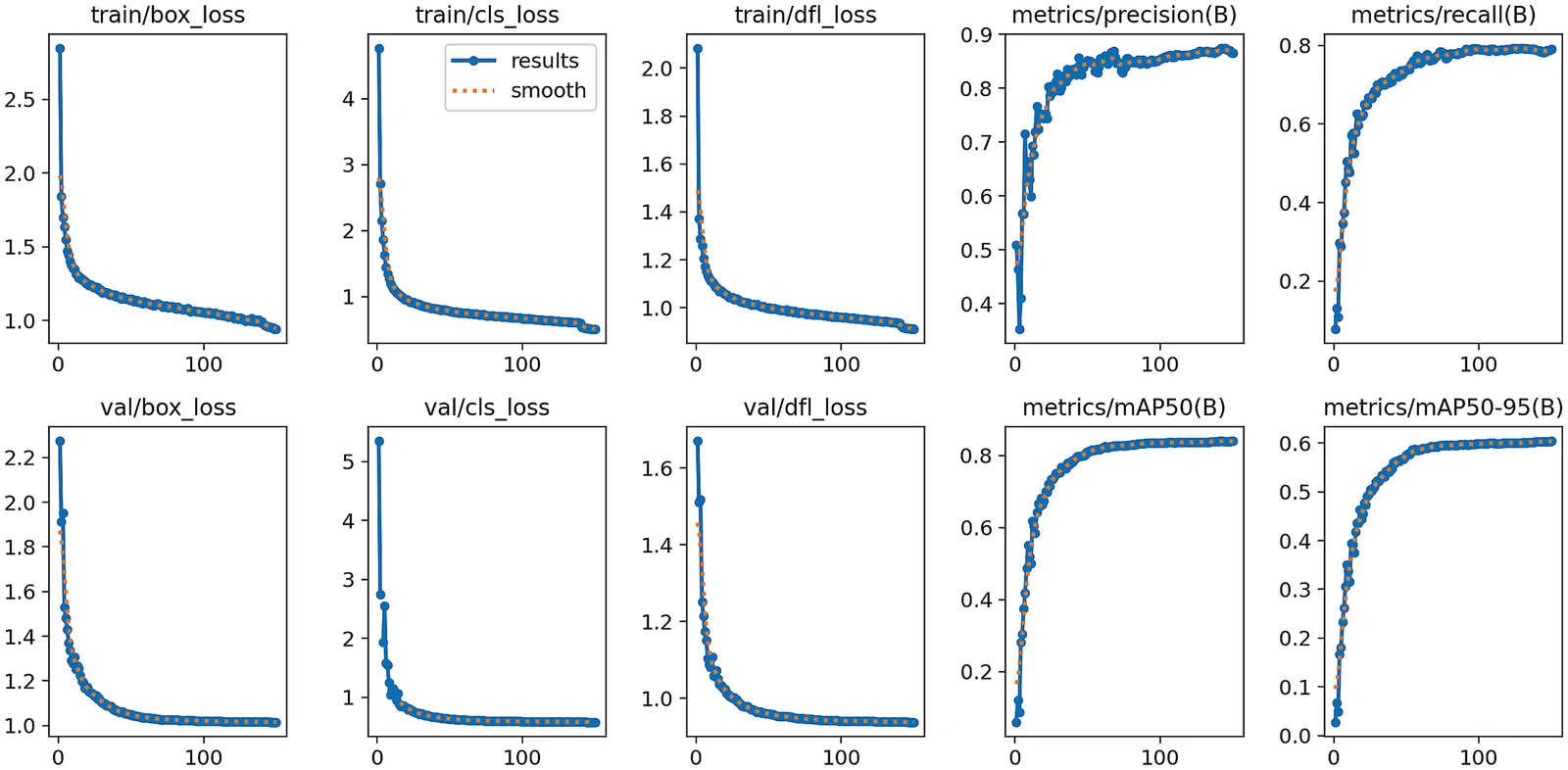
GFLNet training results on UCAS-AOD dataset.

### 4.3 Evaluation metrics

The evaluation includes two aspects: model complexity and detection accuracy. Complexity is measured using Params and GFLOPs. Detection accuracy is typically assessed using Precision (P), Recall (R), and mAP. In view of the focus of this study on small target detection, AP_S and AP_L are additionally reported in the ablation experiment.


$$\begin{array}{c} Precision=\frac{TP}{TP+FP} \end{array}$$ (21)

$$\begin{array}{c} Recall=\frac{TP}{TP+FN} \end{array}$$ (22)

$$\begin{array}{c} AP=\int_{\text{0}}^{\text{1}}P\left(R\right)dR \end{array}$$ (23)

$$\begin{array}{c} mAP=\frac{\sum_{i=1}^{N}AP_{i}}{N} \end{array}$$ (24)

Here, ***TP*** denotes correctly detected positive instances, ***FP*** denotes false-positive detections, ***FN*** denotes missed positive instances, and N is the total number of instances in the target category.

### 4.4 Comparison experiments

Experiments were conducted on DIOR and UCAS-AOD to compare GFLNet with representative two-stage, one-stage and lightweight detectors. On DIOR, GFLNet is compared with Faster R-CNN [3], Mask R-CNN [4], YOLOv5 [5], YOLOv8 [6], YOLOv10 [35], YOLOP [36], YOLO-TLA [16], and YOLO11n. Recent lightweight detectors, including GSS-YOLO [37], Dynamic-3T-KD [38], and PR-YOLO [39], are also included for comparison. As summarized in Table 1, GFLNet has the best mAP@0.5 among all evaluated methods, reaching 84.9%, with only 2.35M parameters and 5.6 GFLOPs. This indicates that high detection accuracy does not necessarily require a large computational budget. For a finer-grained comparison of small-object detection, GFLNet achieves an AP_S of 59.2%, showing strong small-object detection capability while maintaining low model complexity.

**Table 1:** Comparison on DIOR dataset (%).

Model	airplane	ship	bridge	dam	vehicle	harbor	airport	chimney	mAP@0.5	AP_S
Faster-RCNN [3]	88.1	77.3	81.2	72.3	84.5	75.4	79.5	74.7	79.4	–
YOLOv5 [5]	87.8	76.9	80.6	71.5	83.1	74.1	78.3	73.5	78.5	–
YOLOv8 [6]	89.4	78.4	82.3	73.6	85.3	76.3	80.6	75.8	80.2	–
YOLOv10 [35]	89.2	79.2	82.3	74.3	86.2	77.0	81.0	76.4	80.9	–
Mask-RCNN [4]	88.3	78.1	81.4	70.9	85.0	75.7	80.0	75.3	79.7	–
YOLOP [36]	87.1	75.3	79.1	70.8	82.4	73.3	79.5	72.6	77.3	–
YOLO11n [7]	90.3	79.5	83.3	74.7	86.5	77.9	81.9	77.2	81.0	79.2
GSS-YOLO [37]	94.3	87.6	57.0	72.5	59.8	65.8	79.8	93.1	82.2	80.5
3T-KD [38]	93.1	94.5	50.2	73.2	75.2	74.1	86.7	91.1	80.4	77.6
PR-YOLO [39]	91.7	81.7	85.1	76.6	88.2	79.4	83.4	79.1	83.7	81.4
YOLO-TLA [16]	90.2	82.3	84.2	74.6	88.5	78.4	82.7	77.8	82.8	79.8
Ours	92.1	82.6	82.6	77.2	89.1	80.3	84.3	79.9	**84.9**	82.3

This result benefits from the collaborative design of three modules. GSD-Fuse strengthens the interaction between spatial and channel features with low computational overhead. Inspired by LSKNet, the receptive-field selection module adapts the contextual range to local image content. This improves sensitivity to small objects and scale changes. LB-Compress integrates LAMP pruning with BCKD distillation. It reduces the model size while recovering accuracy lost during compression. GSD-Fuse improves feature representation, LSK adjusts context modeling according to the target scale, while LB-Compress maintains accuracy after compression. Therefore, they complement each other and jointly improve localization and classification performance. The mAP@0.5 results, which jointly reflect precision and recall, confirm that GFLNet achieves a strong accuracy-efficiency trade-off and is suitable for remote sensing deployment under limited resources.

On UCAS-AOD, GFLNet is compared with NAS-FCOS [40], RT-DETR [41], Zhang et al. [42], Li et al. [43], LPEC-RDet [44], LMSD-YOLO [17], and common YOLO baselines. As shown in Table 2, GFLNet’s mAP@0.5 reaches 96.9%, while its mAP@0.5:0.95 reaches 75.4%, both exceeding the corresponding results of all comparison methods, including the baseline model YOLO11n (93.5% and 72.3% respectively). Furthermore, GFLNet compares favorably with recent lightweight remote sensing detectors such as LPFC-RDet. Compared with recent lightweight models such as YOLOv10 and YOLOv8, the number of GFLNet parameters is only 2.35M and the computational cost is 5.6 GFLOPs, but the detection accuracy is higher. This result indicates that the proposed improvement does not sacrifice architectural efficiency while improving accuracy. Compared with anchor-free and rotation-aware models such as LMSD-YOLO and Li et al, GFLNet has also achieved a better balance between accuracy and resource consumption.

**Table 2:** Comparison on UCAS-AOD dataset (%).

Model	mAP@0.5	mAP@0.5:0.95	Params(M)	GFLOPs	FPS
Faster-RCNN [3]	88.36	70.47	31.5	22.7	15
YOLOv5 [5]	90.13	72.54	8.24	31.4	95
YOLOv8 [6]	91.69	73.79	9.67	27.5	100
YOLOv10 [35]	92.51	75.12	7.58	21.9	150
NAS-FCOS [40]	89.44	70.90	34.20	25.6	12
RT-DETR [41]	89.87	71.23	41.27	80.2	108
Zhang et al. [42]	92.45	74.75	14.6	27.8	72
Li et al. [43]	93.68	74.98	15.9	29.3	86
LPFC-RDet [44]	96.8	74.55	2.22	22.3	47
LMSD-YOLO [17]	92.5	75.1	13.2	24.7	98
YOLO11n [7]	93.5	72.3	3.28	6.5	199
Ours	**96.9**	75.4	**2.35**	**5.6**	261

To further evaluate practical deployment efficiency, YOLO11n and GFLNet were tested on an NVIDIA Jetson AGX Xavier. The same input size and inference settings were used for both models. The results are shown in Table 3.

**Table 3:** Inference performance on the embedded edge platform.

Model	Params(M)	GFLOPs	Params(M)	Latency(ms)	FPS
YOLO11n	3.28	6.5	3.28	5.03	199
GFLNet	**2.35**	**5.6**	**2.35**	3.83	261

GFLNet achieves a lower inference latency than YOLO11n on the embedded platform. This result further confirms the deployment efficiency of the proposed lightweight design.

Taken together, the results on both benchmarks validate the effectiveness of GSD-Fuse, the LSKNet-inspired receptive-field selection module, and LB-Compress as a coordinated detection framework. GFLNet achieves a consistent accuracy-efficiency balance in different remote sensing scenarios.

### 4.5 Ablation study

We conducted ablation studies on DIOR and UCAS-AOD to assess the effect of each proposed component. YOLO11n served as the baseline. All experiments used the same training settings to ensure a fair comparison. LSK and GSD-Fuse were then added in stages to evaluate their separate and combined contributions. As shown in Table 3 and 4, the added modules consistently improve several evaluation metrics, with a low computational burden.

**Table 4:** Ablation experiment on DIOR dataset.

Method	P	R	mAP@0.5	mAP@0.5:0.95	AP_S	AP_L	Param(M)	GFLOPs
**YOLO11n (A)**	84.1	75.5	81.0	60.0	55.8	66.9	3.28	6.5
**A + LSK (B)**	84.6	76.5	82.9	61.7	57.4	68.1	3.26	6.6
**B + GSConv**	84.9	77.0	83.2	62.5	58.3	68.8	3.25	6.5
**B + GSD-Fuse** **(GFL-YOLO)**	85.7	77.8	83.7	63.3	59.2	69.4	3.24	6.3

Table 4 details the ablation study on DIOR. The baseline achieves 81.0% mAP@0.5 with 3.28M parameters. When the LSK module is introduced alone, the model attains 84.6% precision and 76.5% recall. Meanwhile, the parameter counts slightly decrease to 3.26M, and the GFLOPs only raises by 0.1. This shows that adaptive receptive-field selection can improve the contextual feature extraction without much computational cost. Besides, mAP@0.5 increases to 83.7% when GSD-Fuse is further added, and the recall increases to 77.2%. Moreover, the parameter counts and GFLOPs are reduced to 3.24M and 6.3M, respectively. Compared with AP_L, the improvement in AP_S is more obvious, which implies that more feature interaction and context selection will be helpful for small objects.

To further evaluate the performance improvement of GFL-YOLO, it is compared with the YOLO11n baseline on DIOR. The PR curves of the two models are illustrated in Fig 10. GFL-YOLO has been consistently better than YOL11n in terms of detection performance. This confirms the effectiveness of the proposed structures for detection. To further isolate the contribution of GSD-Fuse, GSConv was inserted at the same positions under the same training settings. Compared with GSConv, GSD-Fuse improves mAP@0.5 by 0.5% and AP_S by 0.9%, while the parameter counts and GFLOPs remain close.

**Fig 10:**
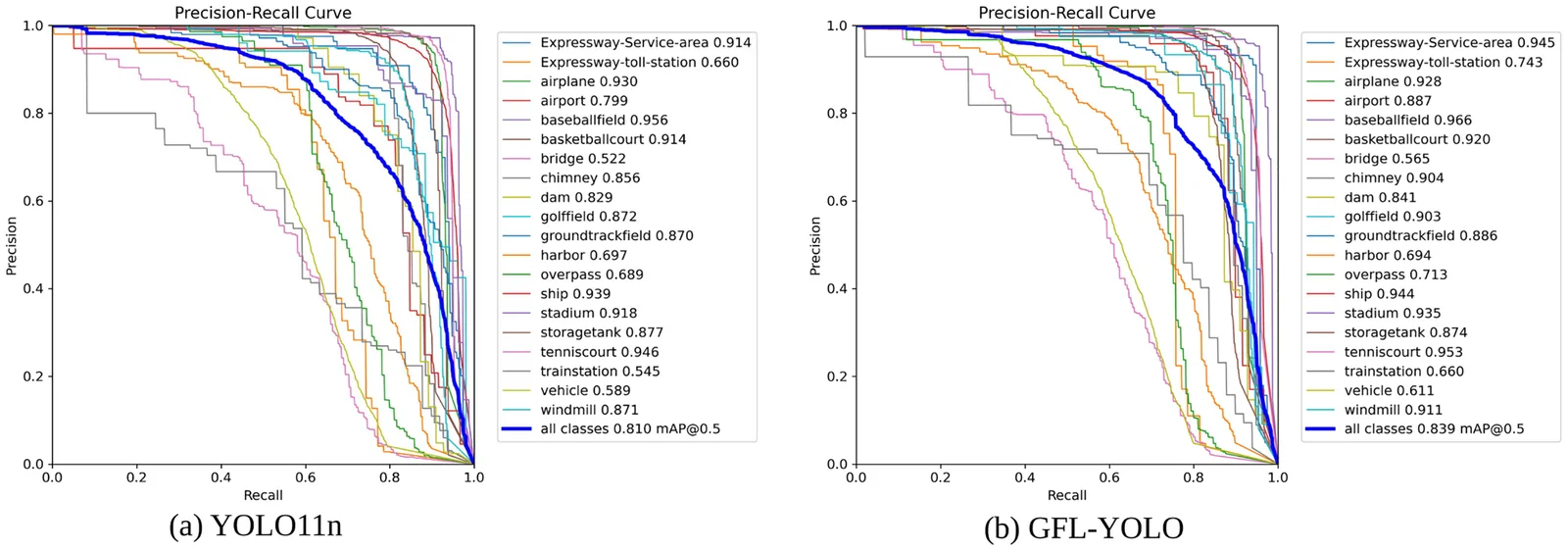
Precision–recall curves on DIOR (YOLO11n vs. GFL-YOLO).

From the category-level results, GFL-YOLO is more effective across all 20 object classes. The most obvious gain is in the train station category, GFL-YOLO is better than YOOL11n by 11.5%. These results show that the model can deal with difficult and underrepresented categories more effectively. This is mainly due to stronger feature extraction and context modelling capabilities which provide more discriminant information.

Table 5 reports the ablation finding on UCAS-AOD. The mAP@0.5 of the baseline model is 93.5%. mAP@0.5 with GSD-Fuse increased to 94.6% and mAp@0.5 without GSDFuse to 74.7%. After combining the two modules, GFL-YOLO achieved the best results: mAP@0.5 was 96.8%, the precision was 94.6%, and the recall was 95.5%. This is 3.3% higher than the baseline model and exceeds the gain brought by the use of any module alone. GSD-Fuse models space and channel information with a compact structure, while LSK adjusts the context range according to the target scale. These two components play a complementary role, which explains why the integrated model can achieve stronger performance.

**Table 5:** Ablation experiment on UCAS-AOD dataset.

Metrics	Baseline	+GSD-Fuse	+LSK	GFL-YOLO
**P**	93.8	94.2	94.0	94.6
**R**	93.1	93.9	93.5	95.5
**mAP@0.5**	93.5	94.6	94.7	96.8
**mAP@0.5:0.95**	72.3	73.1	73.8	75.1

### 4.6 Pruning and distillation analysis

#### 4.6.1 Pruning analysis.

We evaluated GFL-YOLO at different levels of pruning. The speed ratio indicates the pruning strength, 1.0 represents the original model, and larger values indicate heavier compression. Each pruning model is fine-tuned to restore the lost accuracy.

Table 6 reports the results at six compression levels. Moderate pruning will slightly improve accuracy, but stronger pruning will lead to reduced performance. The best equilibrium point occurs when the speed ratio is 1.8. At this time, GFL-YOLO reaches 83.9% mAP@0.5 under the condition that the parameter count is 2.35 million, the model size is 5.2 MB, and the calculation cost is 5.6 GFLOPs. At a speed ratio of 1.8, the learnable threshold achieves 0.7% higher mAP@0.5 than the fixed threshold with nearly unchanged inference efficiency. Increasing the speed ratio from 1.8 to 2.4 only improves FPS from 261 to 273, while mAP@0.5 decreases from 83.9% to 82.7%. Therefore, a speed ratio of 1.8 provides a better trade-off between detection accuracy and computational efficiency.

**Table 6:** Pruning result at different speed-up levels.

Speed	Threshold strategy	mAP@0.5	Params/M	GFLOPs	Model Size/MB	FPS
1.0	Learnable	83.7	3.24	6.3	9.5	199
1.6	Learnable	84.2	2.52	5.8	6.1	258
1.8	Fixed	83.2	2.35	5.6	5.2	260
**1.8**	Learnable	**83.9**	**2.35**	**5.6**	**5.2**	**261**
2.0	Learnable	83.6	2.26	5.1	5.0	267
2.2	Learnable	83.2	2.24	5.0	4.9	271
2.4	Learnable	82.7	2.21	4.9	4.6	273

Based on the results, we chose a speed ratio of 1.8 for GFL-YOLO. Under this setting, the model stays accurate performance while becoming much smaller and more computation-efficient.

#### 4.6.2 Distillation analysis.

The GFL-YOLO model with a speed ratio of 1.8 is used as the student network, and SWL-YOLO11s [45] is used as the teacher network. The experiment compared logit-based methods, including L1, L2 and BCKD, as well as feature-based methods, such as CWD, MGD and CHSIM. Table 7 reports the comparison of distillation methods on DIOR.

**Table 7:** Comparison of distillation methods on the DIOR dataset.

Type	Method	P	R	mAP@0.5	mAP@0.5:0.95
**Logit**	**L1**	83.1	76.3	84.2	64.5
	**L2**	84.2	**78.0**	84.7	64.3
	**BCKD**	**84.7**	77.8	**84.9**	**64.5**
**Feature**	**CWD**	82.4	76.5	83.6	61.7
	**MGD**	82.5	77.3	83.1	61.2
	**CHSIM**	83.4	77.1	82.8	62.3

In the logit-based methods, BCKD achieves the best performance, with 84.7% precision, 77.8% recall, 84.9% mAP@0.5. L2 obtains a slightly better recall of 78.0% but lower values on the other metrics. Although CWD is the most stable feature-based method, its mAP@0.5 is 1.3% lower than those of BCKD. MGD and CHSIM exhibit slightly inferior performance across all metrics, indicating that the feature distillation methods adopted in this study have limitations in transferring feature representations from the teacher model.

All distillation methods use the same pruned student architecture, so they introduce no additional parameters or FLOPs during inference. Overall, BCKD provides the highest mAP@0.5 without increasing the inference complexity. Therefore, BCKD is selected as the final distillation strategy to preserve detection capability.

The sensitivity of the main compression hyperparameters was also examined. As shown in Table 6, a speed ratio of 1.8 provides a favorable balance between accuracy and efficiency, while stronger pruning gradually reduces mAP. For the distillation loss, different weighting ratios between the classification and localization terms were tested. A balanced setting produced the best overall performance, whereas excessive emphasis on either term caused a slight performance decrease. Therefore, the final hyperparameters were selected based on this accuracy-efficiency balance.

Table 8 presents a progressive analysis of each design stage and shows the cumulative gain of the proposed strategy relative to the YOLO11n baseline. The structural version of GFL-YOLO is obtained by embedding the GSD-Fuse and LSKNet-inspired receptive-field selection module in YOLO11n. After this adjustment, it achieves a significant improvement in detection accuracy: the mAP@0.5 increase by 2.7%. Both parameters and model size are slightly reduced. Meanwhile, the computational complexity decreases to 6.3 GFLOPs, further supporting practical deployment.

**Table 8:** Pruning result at different speed-up levels.

Model	mAP@0.5	mAP@0.5:0.95	Params/M	GFLOPs	Model Size	FPS
YOLO11n	81.0	60.0	3.28	6.5	9.5	199
GFL-YOLO	83.7	63.3	3.24	6.3	8.3	204
GFL-YOLO+ pruning	83.9	63.6	2.35	5.6	5.2	261
GFLNet	84.9	64.5	2.35	5.6	5.2	261

To address the issue of increased computational complexity, layer-adaptive channel pruning and knowledge distillation are further applied to GFL-YOLO. Compared with YOLO11n, GFLNet decreases parameter count, computational cost, and model size by approximately 28%, 14%, and 45%, respectively. The mAP@0.5 of GFLNet reaches 84.9%, representing a further performance improvement.

Fig 11 shows the training curves of YOLO11n, GFL-YOLO and GFLNet models over 150 epochs. In the early training stage, GFLNet starts from a slightly higher value, indicating that the structural improvement provides certain initialization advantages. During the middle stage of training (epochs 50–125), GFLNet shows the clearest advantage in mAP@0.5:0.95, suggesting that adaptive receptive-field selection gradually improves multi-scale localization. In the later stage of training (epochs 125–150), the curve becomes stable. GFLNet finally reaches 84.9% mAP@0.5, which is 3.9% higher than YOLO11n. The larger gain in mAP@0.5:0.95 indicates that localization accuracy can still be maintained after pruning and distillation under stricter IoU thresholds. In addition, the curves of GFL-YOLO and GFLNet remain close to each other and are clearly separated from YOLO11n, which shows that LB-Compress substantially reduces the parameter count while causing only minor performance loss.

**Fig 11:**
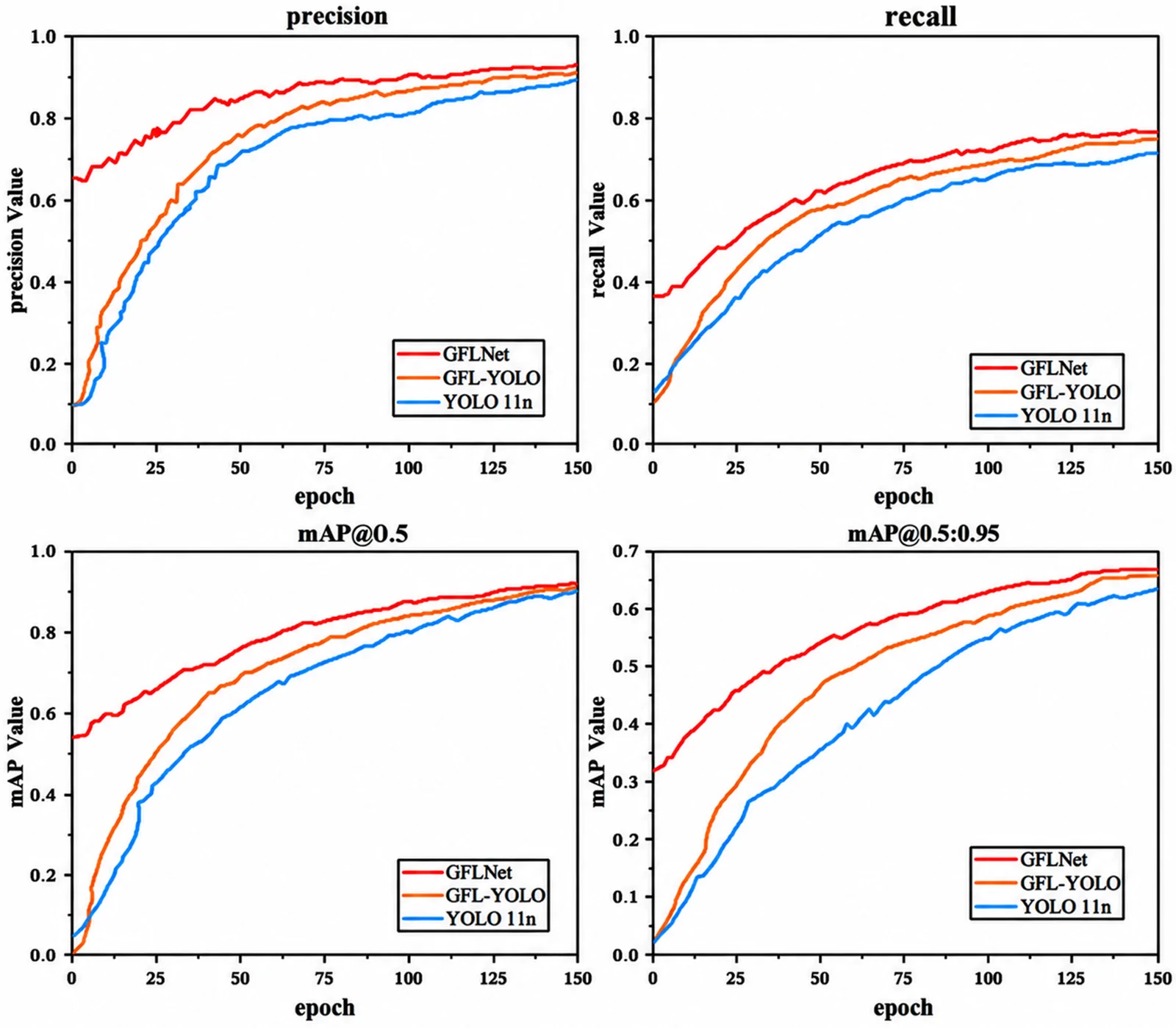
Training curves on DIOR dataset.

In summary, structural redesign, pruning and distillation perform complementary roles: GSD-Fuse and LSK modules improve representation quality, LAMP pruning achieves lightweight, and BCKD distillation compensates for information loss. In the end, GFLNet is suitable for real-time RSOD under limited-resource settings.

### 4.7 Visualization

To verify the GFLNet algorithm on remote sensing objects, we select typical samples from DIOR dataset for qualitative analysis and compare them with the YOLO11n baseline. Fig 12 shows the visualization of the detection results. In scenes with densely packed objects, YOLO11n tends to produce both false positives and missed detections. GFLNet, by contrast, reduces such failures, identifying and classifying many densely spaced objects. Under complex background, YOLO11n frequently misses ships and storage tanks below 30 × 30 pixels. In contrast, GFLNet captures local features of the small targets by dynamically adapting the receptive field of its LSK module and the multi-scale attention mechanism of GSD-Fuse. When it comes to large objects, the baseline also underperforms relative to the enhanced model. Overall, the qualitative results confirm that GFLNet maintains robust and consistent detection across diverse scenarios.

**Fig 12:**
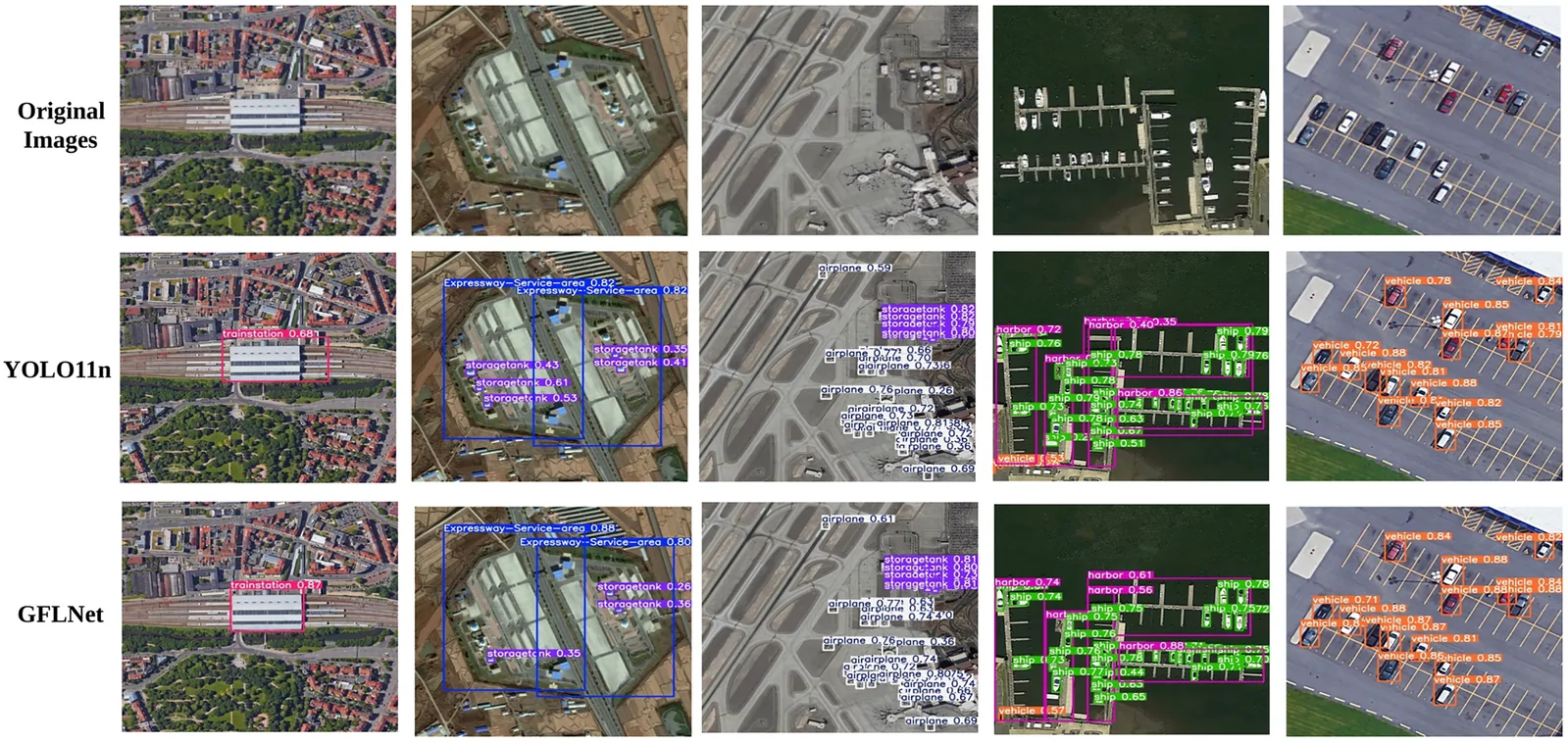
Detection visualization.

Additional qualitative detection results are shown in Fig 13 using representative images from VisDrone Dataset. These images contain challenging conditions such as low-light, overcast/rainy weather, and strong illumination. The results show that GFLNet can still produce reliable detections in these scenes, which suggests good robustness under varying visual conditions. Overall, the qualitative results in Figs 12 and 13 show that GFLNet maintains reliable detection performance across diverse and challenging scenes.

**Fig 13:**
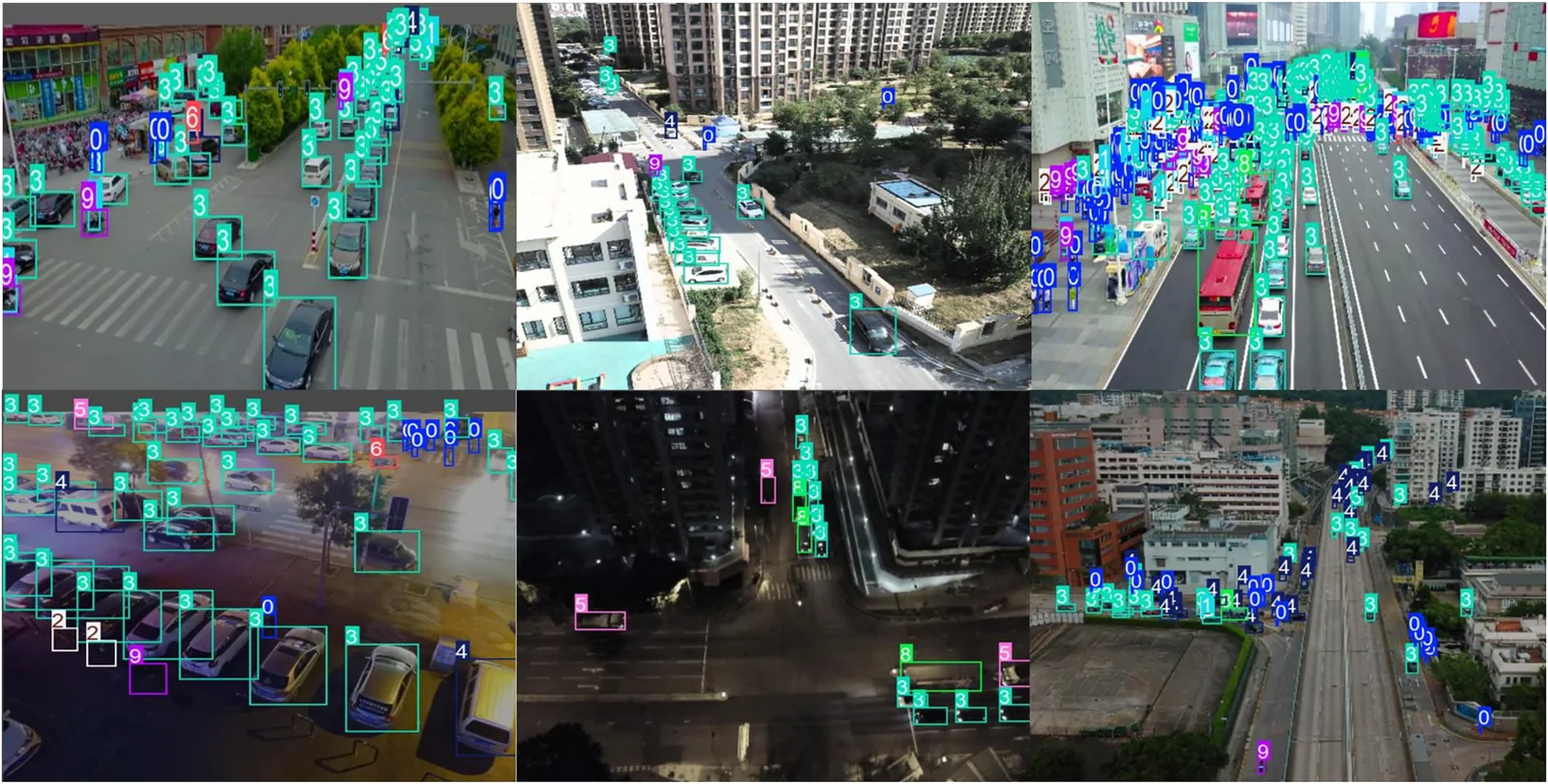
Qualitative detection results of GFLNet on challenging scenes from the VisDrone-2019 dataset. The source images were obtained from the VisDrone-2019 dataset deposited on Figshare (https://doi.org/10.6084/m9.figshare.28683947.v1), licensed under CC BY 4.0.

## 5 Conclusions

In this paper, we propose GFLNet, a lightweight detector for RSOD. The proposed framework combines GSD-Fuse for spatial channel feature interaction, LSK for content adaptive receptive field selection, LB-Compress for compressive sensing model optimization. With this design, GFLNet enhances the feature representation for dense small targets and targets with large scale variations, with low computational cost. In experiments on DIOR and UCAS-AOD, GFLNet has been proved to be effective in small target detection, scale adaptation, and post compression accuracy. Ablation studies showed that the combination of GSD-Fuse and LSK has complementary effects. LB-Compress also reduces the model size without sacrificing detection performance. The visualization results show that GFLNet produces fewer missed detections and more accurate bounding boxes. It also generates stronger feature responses in complex scenes. These results support its use on resource-constrained devices. However, the current experiments use only RGB images. Its robustness under low-visibility conditions still requires further evaluation. Future work will investigate multimodal data, domain adaptation, and self-supervised learning to improve generalization in real-world applications.
